# Modeling distress in cancer patients using a fuzzy logic approach based on expert consensus

**DOI:** 10.1186/s12957-026-04431-2

**Published:** 2026-05-30

**Authors:** Anushk Pandey, Bejoy C Thomas, Manoj Pandey

**Affiliations:** 1 Department of Computer science, Vellore Institute of Technology, Vellore, India; 2 Department of Computer Sciences, Tandon College of Engineering, New York University, Brooklyn, NY 11201 USA; 3 Division of Psychosocial Oncology, Oncology, Cumming School of Medicine, University of Calgary, Calgary, Canada; 4 Department of Surgical Oncology, Institute of Medical Sciences, Banaras Hindu University, Varanasi, 221005 India; 5 Resource centre, Health Technology Assessment India, Banaras Hindu University, Varanasi, 221005 India

**Keywords:** Distress, Cancer, Psychosocial oncology, Fuzzy Logic, Delphi Process, System Dynamics

## Abstract

**Objective:**

To develop and validate a fuzzy logic model based on expert consensus to elucidate distress dynamics in cancer patients, examining the non-linear interactions between psychological, social, and medical factors.

**Methods:**

A two-round Delphi process with 23 psychosocial oncology experts was conducted to generate an interaction matrix of 18 distress-related variables. Using the *skfuzzy* Python library, a Mamdani fuzzy inference system was constructed, focusing on Negative Psychological Factors, Symptoms, and Positive Psychological Factors as primary drivers. Model validation included network analysis, time-series simulations, and sensitivity analyses, compared against a traditional crisp system dynamics model.

**Results:**

The fuzzy model confirmed a self-reinforcing “vicious cycle” of distress driven by Negative Psychological Factors (weight = 2.00) and Symptoms (weight = 1.50). Simulations demonstrated that positive psychological interventions could reduce overall distress levels by up to 25%. Network analysis identified distress as a central system hub, while the fuzzy model produced smoother, more clinically realistic trajectories than the crisp model.

**Conclusion:**

This study provides a robust mathematical explanation for the success of the validated DIC-2 clinical tool. The results underscore the necessity of early, multidisciplinary interventions to disrupt distress cycles, supporting the clinical shift toward treating distress as the “sixth vital sign”.

**Graphical Abstract:**

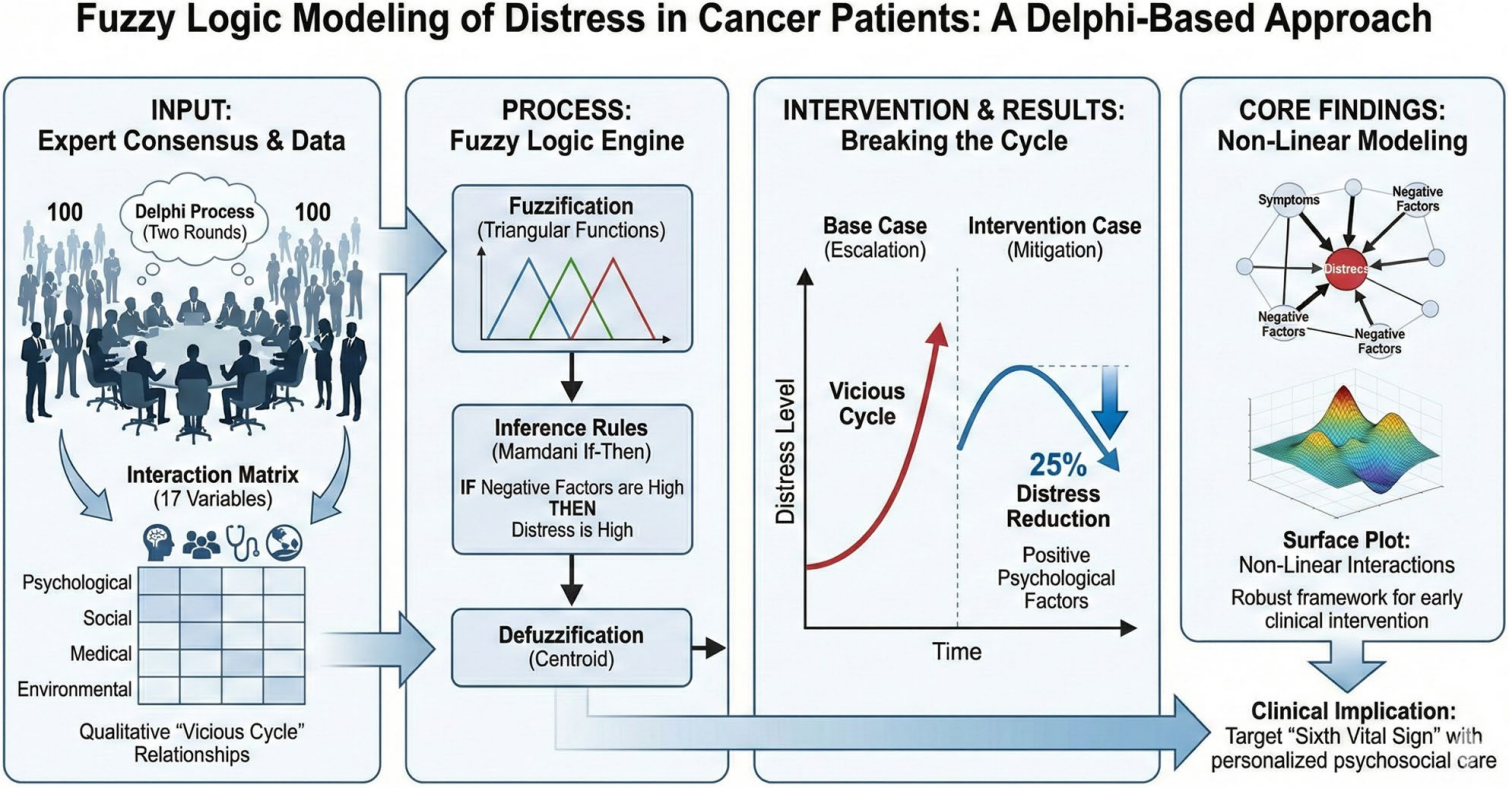

## Introduction

Distress in cancer patients, defined as “an unpleasant emotional experience of a psychological, social, and/or spiritual nature that interferes with the ability to cope effectively with cancer and its treatment” [1], is a critical challenge in psychosocial oncology. It manifests along a continuum, ranging from normal feelings of sadness to debilitating conditions like depression and anxiety [1, 2]. Beyond emotional suffering, it serves as a significant predictor of treatment response and survival. The complexity of psychological distress arises from its interplay with equally complex biological, social, and environmental factors [3–7]. Furthermore, distress has been shown to overlap with anxiety and depression [8], alter the quality of life [9] and can be a predictor of response, survival and follow-up in patients with cancer [10, 11]. Understanding these interactions is essential for developing effective interventions to mitigate distress and improve patient outcomes.

While contemporary tools like the NCCN Distress Thermometer offer rapid clinical screening, they are inherently linear and provide only a static snapshot of a patient’s state [5, 6, 12–14]. Computational approaches, such as system dynamics and network analysis, have also been employed to capture the dynamic interplay of distress-related factors [15, 16]. The current work addresses a critical gap by providing a non-linear, dynamic framework capable of modeling the complex interplay between psychological, social, and medical factors. This study represents a computational re-evaluation of the foundational 1998–2000 Delphi consensus that served as the blueprint for the Distress Inventory for Cancer (DIC) -2. Fuzzy logic is uniquely suited for modeling such subjective phenomena, as it effectively handles qualitative uncertainty and expert-driven nuances [17, 18]. Furthermore, when empirical data is limited, the Delphi method provides a robust, consensus-building approach to map complex variable relationships [19, 20]. By utilizing a Mamdani Fuzzy Inference System (FIS), we move beyond simple correlations to simulate the ‘vicious cycle’ of distress, offering a robust methodology that handles the linguistic ambiguity inherent in clinical expert judgment.

We hypothesized that distress forms a self-reinforcing “vicious cycle” driven by negative factors, which can be mitigated by positive psychological, social, and environmental interventions. Our specific objectives were to: (1) develop a distress model based on expert consensus, (2) quantify the influence of key variables, and (3) Visualize the dynamic behavior of distress through network graphs, time series simulations, and sensitivity analyses.

## Methods

### Delphi process and data collection

The foundational data for this study was gathered through a structured two-round Delphi process designed to identify dyadic relationships between variables hypothesized to influence distress in cancer patients. We identified 100 international experts in psychosocial oncology by cross-referencing membership registers of professional associations with PubMed citations of which 23 responded to invitation. In the initial round, experts evaluated an interaction matrix comprising 14 variables (Table 1) that including negative psychological factors, symptoms, and diagnosis using a 6-point qualitative scale ranging from strong positive (++) to strong negative (– –).

**Table 1:** Expert opinion-based interaction matrix of distress (Round 1 consensus) This matrix displays the initial dyadic relationships between 14 variables hypothesized to influence distress, using a 6-point qualitative scale ranging from strong positive (++) to strong negative (– –)

	Negative Psychological factors	Negative Social factors	Negative Environmental factors	Immune compromise	Carcinogenesis	Symptoms	Distress	Diagnosis	Treatment plan	Patient decision making	Actual treatment	Treatment outcome	Recurrence/ relapse	Time factor
	1	2	3	4	5	6	7	8	9	10	11	12	13	14
**1**	**0.00**	++	0	+	+	++	++	+	+	++	+	+	+	+
**2**	+	**0.00**	+	0	0	+	++	+	+	+	+	+	0	+
**3**	+	+	**0.00**	+	++	0	+	0	+	0	+	0	0	+
**4**	0	0	0	**0.00**	+	+	0	0	+	0	+	+	+	0
**5**	+	+	0	+	**0.00**	+	+	+	++	0	0	+	++	0
**6**	++	+	0	0	0	**0.00**	++	++	++	++	++	0	0	+
**7**	++	+	0	++	+	+	**0.00**	+	+	+	+	+	+	+
**8**	+	+	0	0	0	+	++	**0.00**	++	+	++	+	+	0
**9**	+	+	0	0	0	+	+	0	**0.00**	++	++	++	0	0
**10**	+	+	0	0	0	-	0	0	+	**0.00**	+	+	0	+
**11**	++	+	0	+	0	0	+	0	+	+	**0.00**	++	++	+
**12**	++	+	0	+	0	++	+	0	0	+	+	**0.00**	++	+
**13**	+	+	0	++	0	++	++	+	++	++	++	0	**0.00**	+
**14**	+	+	0	0	0	++	+	+	+	+	+	0	+	**0.00**

**Fig. 1:**
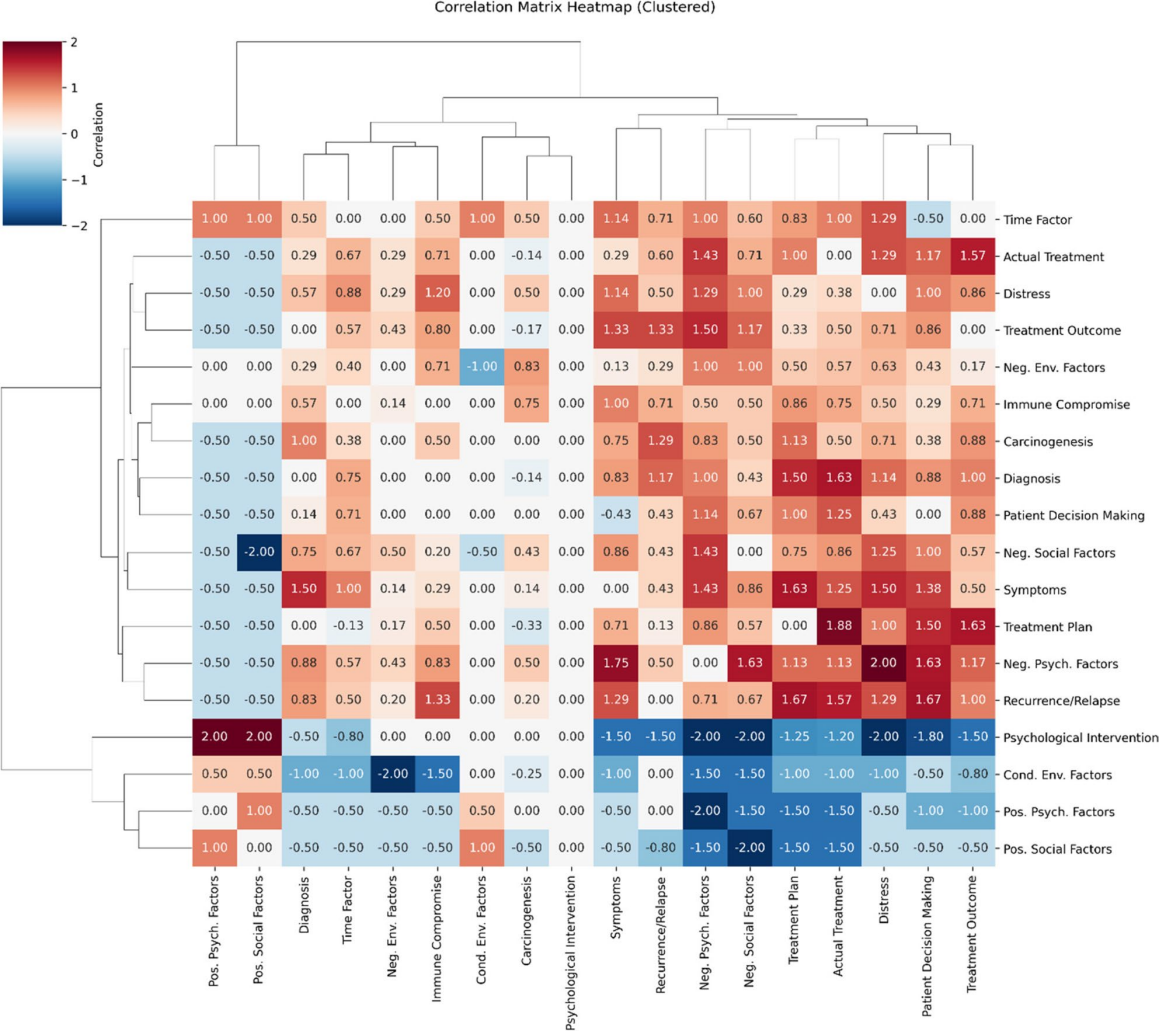
Clustered Correlation Matrix Heatmap of Distress Variables: This heatmap visualizes the interaction weights between 18 distress-related variables derived from the second-round Delphi consensus. Hierarchical clustering is applied to group variables with similar interaction profiles. The color scale represents the strength and direction of influence, ranging from strong inhibitory (dark blue, -2.0) to strong excitatory (dark red, + 2.0)

The second round refined the model by introducing four inhibitory variables: positive psychological factors, positive social factors, conducive environmental factors, and psychological intervention. Psychological Intervention (Variable 18) was modeled as a pure independent input (external actuator). This allows the model to simulate the impact of clinical interventions on the system without assuming that internal distress dynamics reciprocally alter the structural nature of the intervention itself. The transition from the initial 14-variable set to the 18-variable model served as an ablation analysis, demonstrating that the exclusion of inhibitory factors leads to an unstable, escalating distress spiral. Following this round, median consensus scores were calculated from the 8 responses received, to establish the numerical weights used as the basis for the subsequent computational models (Table 2). While a sample size of eight might be considered small for empirical clinical trials, Delphi literature demonstrates that a panel of 5 to 10 highly specialized domain experts is methodologically sufficient to achieve consensus stability when parameterizing structural rule architectures rather than conducting epidemiological polling. To visualize these complex system dynamics and group similar variable interactions, a clustered correlation matrix heatmap was generated (Fig. 1).

**Table 2:** Expert opinion-based interaction matrix of distress after second round consensus This refined matrix includes numerical weights for the original 14 variables plus four inhibitory variables (positive psychological, positive social, conducive environmental, and psychological intervention) (18 rows x 18 column)

	Negative Psychological factors	Negative Social factors	Negative Environmental factors	Immune compromise	Carcinogenesis	Symptoms	Distress	Diagnosis	Treatment plan	Patient decision making	Actual treatment	Treatment outcome	Recurrence/ relapse	Time factor	Positive Psychological factors	Positive Social factors	Conducive Environmental factors	Psychological intervention
	1	2	3	4	5	6	7	8	9	10	11	12	13	14	15	16	17	18
**1**	**0.00**	1.63	0.43	0.83	0.50	1.75	2.00	0.88	1.13	1.63	1.13	1.17	0.50	0.57	-0.50	-0.50	0.00	0.00
**2**	1.43	**0.00**	0.50	0.20	0.43	0.86	1.25	0.75	0.75	1.00	0.86	0.57	0.43	0.67	-0.50	-2.00	-0.50	0.00
**3**	1.00	1.00	**0.00**	0.71	0.83	0.13	0.63	0.29	0.50	0.43	0.57	0.17	0.29	0.40	0.00	0.00	-1.00	0.00
**4**	0.50	0.50	0.14	**0.00**	0.75	1.00	0.50	0.57	0.86	0.29	0.75	0.71	0.71	0.00	0.00	0.00	0.00	0.00
**5**	0.83	0.50	0.00	0.50	**0.00**	0.75	0.71	1.00	1.13	0.38	0.50	0.88	1.29	0.38	-0.50	-0.50	0.00	0.00
**6**	1.43	0.86	0.14	0.29	0.14	**0.00**	1.50	1.50	1.63	1.38	1.25	0.50	0.43	1.00	-0.50	-0.50	0.00	0.00
**7**	1.29	1.00	0.29	1.20	0.50	1.14	**0.00**	0.57	0.29	1.00	0.38	0.86	0.50	0.88	-0.50	-0.50	0.00	0.00
**8**	1.00	0.43	0.00	0.00	-0.14	0.83	1.14	**0.00**	1.50	0.88	1.63	1.00	1.17	0.75	-0.50	-0.50	0.00	0.00
**9**	0.86	0.57	0.17	0.50	-0.33	0.71	1.00	0.00	**0.00**	1.50	1.88	1.63	0.13	-0.13	-0.50	-0.50	0.00	0.00
**10**	1.14	0.67	0.00	0.00	0.00	-0.43	0.43	0.14	1.00	**0.00**	1.25	0.88	0.43	0.71	-0.50	-0.50	0.00	0.00
**11**	1.43	0.71	0.29	0.71	-0.14	0.29	1.29	0.29	1.00	1.17	**0.00**	1.57	0.60	0.67	-0.50	-0.50	0.00	0.00
**12**	1.50	1.17	0.43	0.80	-0.17	1.33	0.71	0.00	0.33	0.86	0.50	**0.00**	1.33	0.57	-0.50	-0.50	0.00	0.00
**13**	0.71	0.67	0.20	1.33	0.20	1.29	1.29	0.83	1.67	1.67	1.57	1.00	**0.00**	0.50	-0.50	-0.50	0.00	0.00
**14**	1.00	0.60	0.00	0.50	0.50	1.14	1.29	0.50	0.83	-0.50	1.00	0.00	0.71	**0.00**	1.00	1.00	1.00	0.00
**15**	-2.00	-1.50	-0.50	-0.50	0.00	-0.50	-0.50	-0.50	-1.50	-1.00	-1.50	-1.00	0.00	-0.50	**0.00**	1.00	0.50	0.00
**16**	-1.50	-2.00	-0.50	-0.50	-0.50	-0.50	-0.50	-0.50	-1.50	-0.50	-1.50	-0.50	-0.80	-0.50	1.00	**0.00**	1.00	0.00
**17**	-1.50	-1.50	-2.00	-1.50	-0.25	-1.00	-1.00	-1.00	-1.00	-0.50	-1.00	-0.80	0.00	-1.00	0.50	0.50	**0.00**	0.00
**18**	-2.00	-2.00	0.00	0.00	0.00	-1.50	-2.00	-0.50	-1.25	-1.80	-1.20	-1.50	-1.50	-0.80	2.00	2.00	0.00	0.00

### Fuzzy logic model

**Fig. 2:**
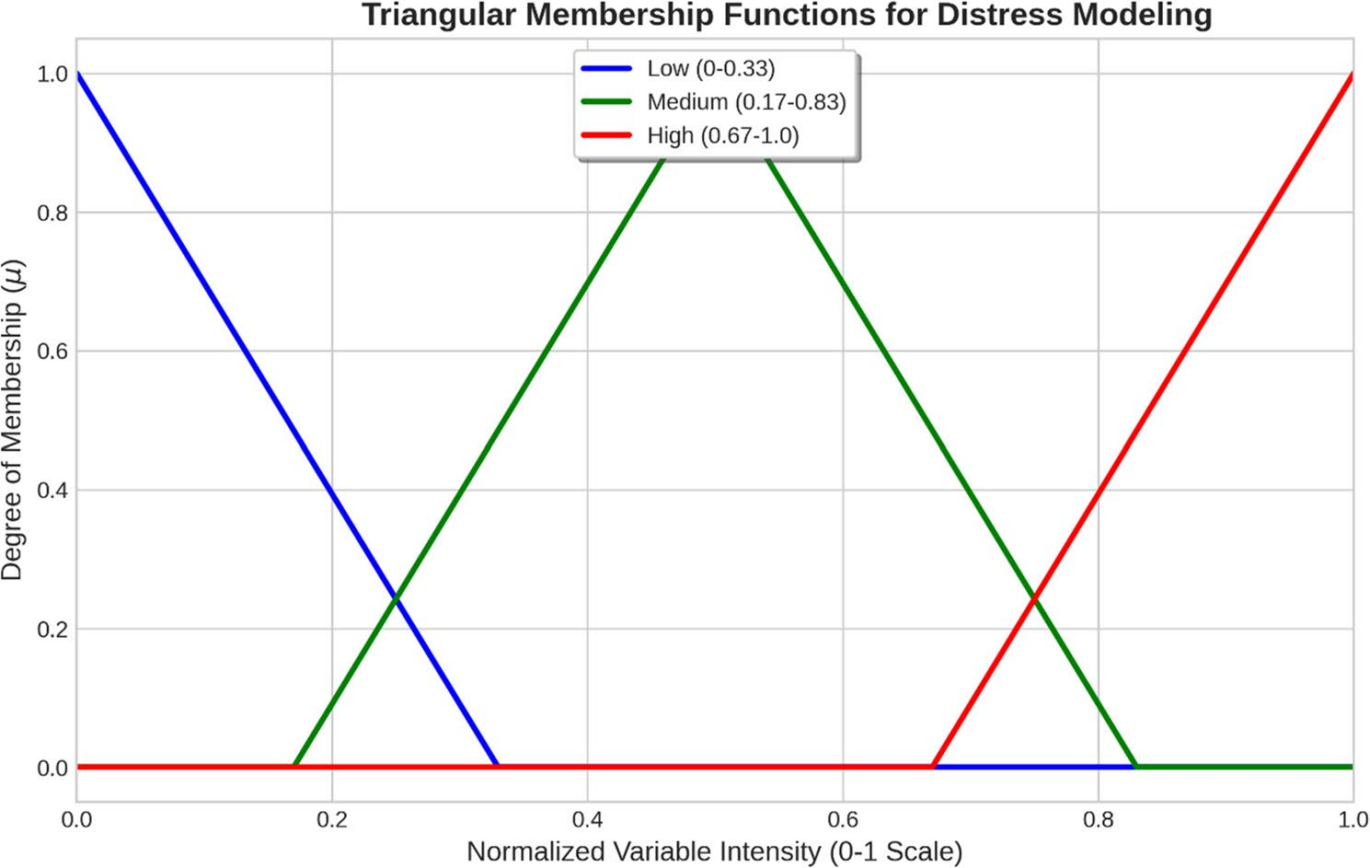
(**A**): Triangular Membership Functions for Distress Modeling Representation of the linguistic categories—Low (0–0.33), Medium (0.17–0.83), and High (0.67–1.0)—used to map crisp input values to the fuzzy logic framework on a normalized 0–1 scale

To address the uncertainty and subjectivity inherent in expert judgments, we implemented a Mamdani Fuzzy Inference System (FIS), a robust mathematical framework for modeling expert knowledge under uncertainty, using the *skfuzzy* library for computational execution. Three primary input variables were selected based on their high absolute influence on distress as determined in the Delphi matrix: Negative Psychological Factors (weight = 2.00), Symptoms (weight = 1.50), and Positive Psychological Factors (weight = -0.50). These variables were mapped to a normalized 0–1 scale using triangular membership functions representing Low (0–0.33), Medium (0.17–0.83), and High (0.67–1.0) states (Fig. 2). For a triangular function defined by parameters (a, b, c), the membership degree µ(x) is calculated as:

$$\mu(x) = \left\{ \begin{array}{ll} 0, & x \leq a \\ \frac{x- a}{b - a}, & a < x \leq b \\ \frac{c-x}{c - b}, & b < x < c \\ 0, & x \geq c \end{array} \right.$$

A Mamdani fuzzy inference system was then implemented, utilizing ten “If-Then” rules derived from the consensus weights (Table 2). The output universe for Distress was defined on a normalized scale of 0 to 1, with the centroid method used to resolve the final crisp value (Table 3).

$${}_{{}_{{\mathit z}_{\mathit C\mathit O\mathit G}\mathit=\frac{\mathit\int{\mathit\mu}_{\mathit A}\mathit{\left(z\right)}\mathit z\mathit d\mathit z}{\mathit\int{\mathit\mu}_{\mathit A}\mathit{\left(z\right)}\mathit z\mathit d\mathit z}}}$$

**Table 3:** Mamdani fuzzy interface system defining the rules derived from distress matrix This table lists the ten “If-Then” rules used to drive the fuzzy inference system, mapping input conditions for Negative Psychological Factors, Symptoms, and Positive Psychological Factors to Distress output levels

Rule ID	IF (Input Condition)	THEN (Output)	Expert Weight
1	Negative Psychological Factors is **High**	Distress is **High**	2.00
2	Symptoms is **High**	Distress is **High**	1.50
3	Positive Psychological Factors is **High**	Distress is **Low**	-0.50
4	Negative Psychological Factors is **Medium**	Distress is **Medium**	1.00
5	Symptoms is **Medium**	Distress is **Medium**	0.75
6	Positive Psychological Factors is **Medium**	Distress is **Medium**	-0.25
7	Negative Psychological Factors is **Low**	Distress is **Low**	0.00
8	Symptoms is **Low**	Distress is **Low**	0.00
9	Positive Psychological Factors is **Low**	Distress is **High**	0.50*
10	**Psychological Intervention is high**	**Distress is Low**	-2.00

### Crisp system dynamics model

For comparison, a crisp system dynamics model was implemented using differential equations. Distress (D) was modeled as:

$$\:\frac{dD}{dt}=\:{\sum\:}_{i\:\ne\:7}{w}_{\left(i7\right){V}_{i}}-\:kD$$

where w(i7) represents the Delphi consensus weights, V_i_ denotes variable intensities (set at 0.5 for negative factors and 0 or 0.5 for interventions), and k = 0.1 serves as a decay rate. This system was solved numerically using *scipy.integrate.odeint*.

### Analysis and visualization

The dynamics of the system were interpreted through several analytical lenses. Network analysis was conducted by constructing a directed graph of the 18 variables, with edges weighted by consensus values to identify central hubs of influence. Time-series simulations were run over 10 arbitrary units to compare distress trajectories in base scenarios against intervention scenarios, where inhibitory factors were introduced at an intensity of 0.5. Finally, a sensitivity analysis was performed by systematically varying Negative and Positive Psychological Factors across intensities of 0.0, 0.5, and 1.0 to quantify their specific impact on the resulting distress levels.

### Statistical analysis

Descriptive statistics (median consensus scores) were used for Matrix in Table 2. Sensitivity analysis varied Negative and Positive Psychological Factors (0.0, 0.5, 1.0) to assess their impact on distress. Because the Mamdani Fuzzy Inference System (FIS) is a deterministic, rule-based computational framework grounded in expert logic, traditional frequentist statistical validation (e.g., p-values) is mathematically inapplicable. Model validation was therefore conducted through structural framework evaluation, boundary-value sensitivity analyses, and comparative stability metrics against an alternative crisp differential equation system.

## Results

### Delphi process outcomes

The final Delphi round involving eight international experts established a predominantly positive interaction matrix, confirming that most studied variables actively contribute to the escalation of patient distress. Key drivers identified with the highest consensus weights (2.00) were Negative Psychological Factors, Symptoms, and Distress itself, indicating a potent, self-reinforcing “vicious cycle”.

The second round introduced inhibitory variables, which provided the negative weights necessary to model distress mitigation. Notable inhibitory weights included Psychological intervention (-2.00), Conducive Environmental Factors (-1.00) and Positive Psychological Factors (-0.50). These findings provided the numerical foundation for the subsequent fuzzy and crisp simulations (Table 4).

**Table 4:** Second round consensus Matrix showing Key weights for distress A summary table identifying the specific numerical weights assigned to each of the 18 variables, highlighting key drivers such as Negative Psychological Factors (2.00) and Symptoms (1.50)

Variable	Weight
Negative Psychological Factors	2.00
Negative Social Factors	1.25
Negative Environmental Factors	0.63
Immune Compromise	0.50
Carcinogenesis	0.71
Symptoms	1.50
Diagnosis	1.14
Treatment Plan	1.00
Patient Decision Making	0.43
Actual Treatment	1.29
Treatment Outcome	0.71
Recurrence/Relapse	1.29
Time Factor	1.29
Positive Psychological Factors	-0.50
Positive Social Factors	-0.50
Conducive Environmental Factors	-1.00
Psychological intervention	-2.00

### Fuzzy logic model

The fuzzy logic model effectively captured the non-linear dynamics of distress by focusing on the primary drivers identified in the Delphi process. In the base case where Negative Psychological Factors and Symptoms were set to moderate levels (0.5) and no interventions were present, distress reached a high output of approximately 0.8 on a 0–1 scale.

Upon the introduction of an intervention, the model demonstrated a significant mitigation effect, reducing the distress level to approximately 0.6. This represents a 25% reduction in overall distress, highlighting the clinical potential of psychological support.

### Crisp model and time series

**Fig. 3:**
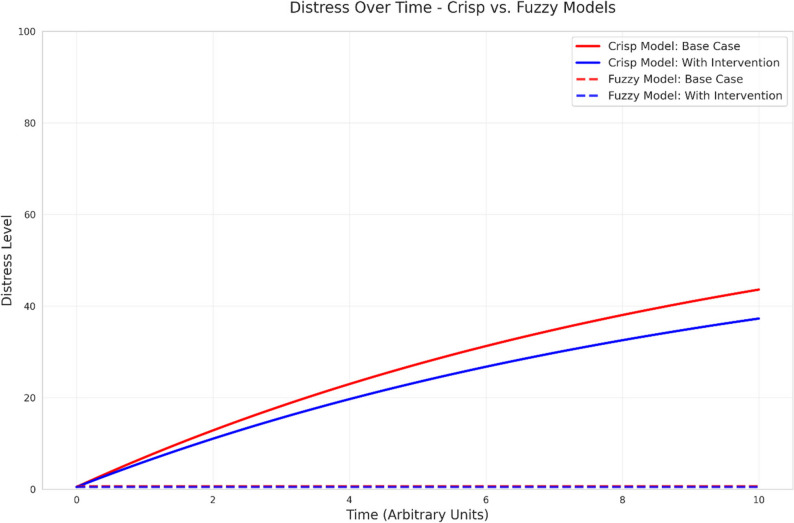
Distress Over Time: Crisp vs. Fuzzy Models: Time series plot showing distress trajectories for base (red) and intervention (blue) scenarios using crisp (solid) and fuzzy (dashed) models. The intervention case shows reduced distress due to inhibitory factors

The crisp system dynamics model, utilizing differential equations, showed distress increasing rapidly from 0.5 to approximately 40 over 10 arbitrary time units in the base case. This rapid growth was attributed to the strong positive weights of negative psychological factors and symptoms. When inhibitory factors were introduced at an intensity of 0.5, the crisp model showed a reduction to approximately 35, translating to a 12.5% reduction in the cumulative distress trajectory (Fig. 3) (Table 5).

**Table 5:** Quantitative Model Comparison

Metric	Crisp Model (ODE)	Fuzzy Model (FIS)
Mitigation Efficiency	12.5% reduction	25% reduction
Steady-State Behavior	Rapid, near-linear growth	Smoother, non-linear saturation
Input Handling	Continuous numerical values	Linguistic categories (Low/Med/High)
Robustness to Uncertainty	Low (requires precise weights)	High (handles expert subjectivity)

As seen in the time-series plots, the fuzzy model produced smoother, more stabilized trajectories compared to the crisp model, which exhibited more extreme peaks. This suggests that the fuzzy logic approach is more robust for modeling the gradual and subjective nature of emotional distress.

### Network analysis

**Fig. 4:**
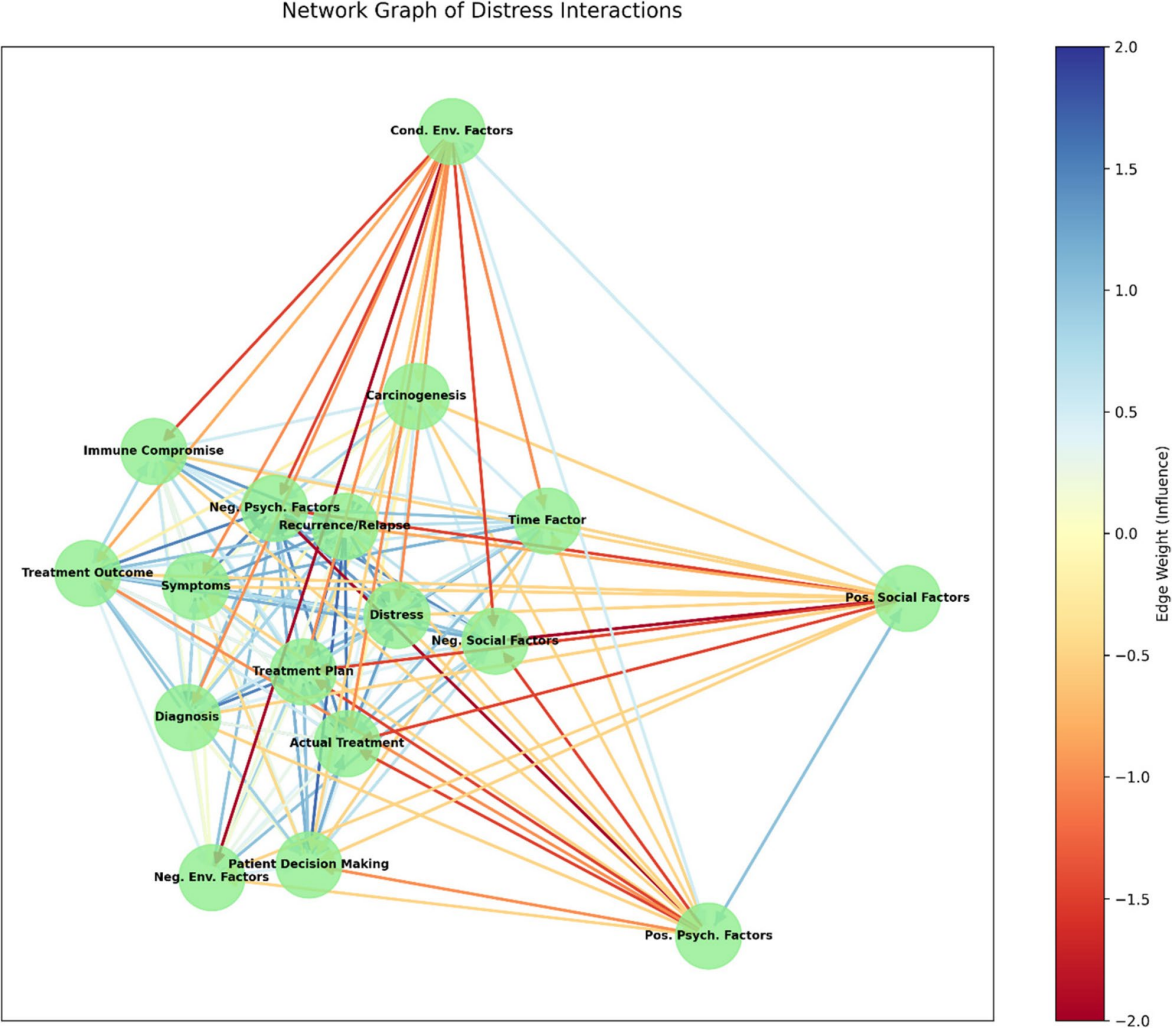
Network Graph of Distress Interactions: Directed graph showing interactions between 18 variables, with edge colors indicating influence direction (blue = positive, red = negative) and weights from Table 2

Network analysis positioned Distress as a central hub in the system, receiving strong positive influences from Negative Psychological Factors and Symptoms, while being negatively influenced by Conducive Environmental Factors and Positive Psychological Factors (Fig. 4).

### Sensitivity analysis

**Fig. 5:**
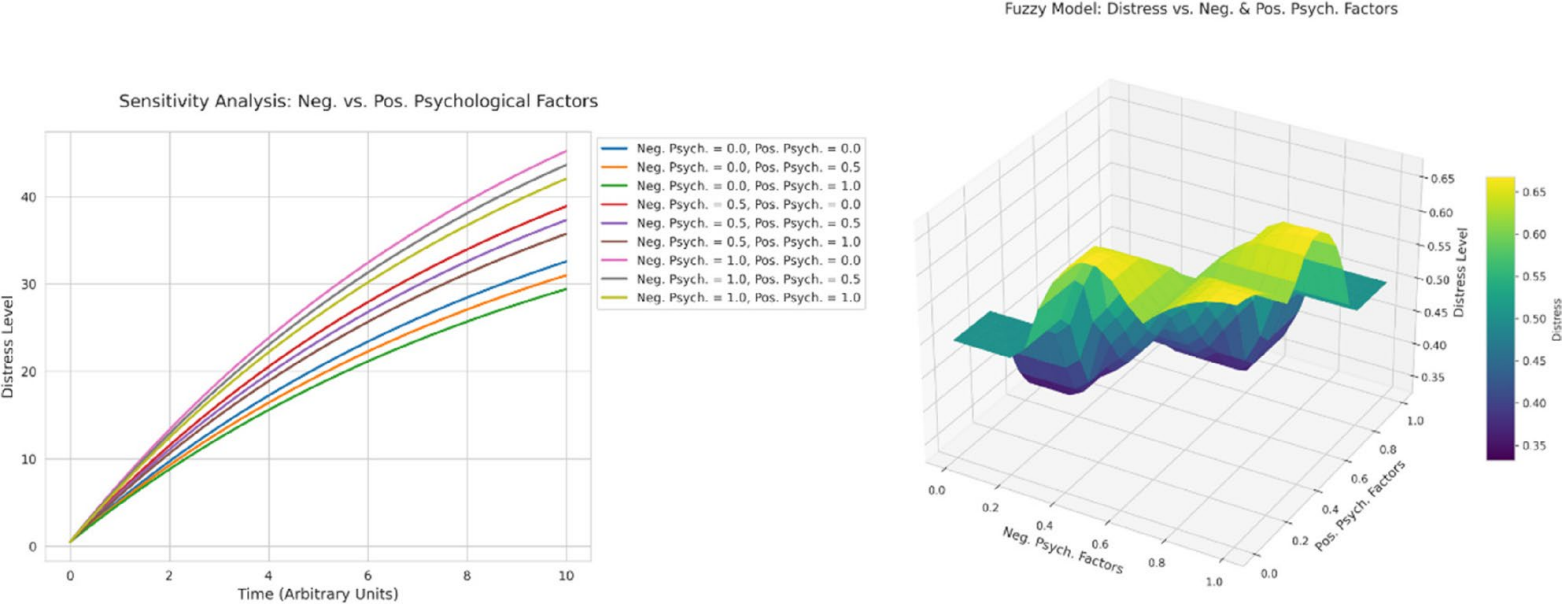
(**A**) Sensitivity Analysis: Neg. vs. Pos. Psychological Factors- Time series showing distress under varying intensities of Negative and Positive Psychological Factors, highlighting the mitigating effect of positive factors. (**B**) Fuzzy Model Surface Plot - Surface plot showing distress as a function of Negative and Positive Psychological Factors (Symptoms fixed at 0.5), demonstrating non-linear interactions

Sensitivity analysis further quantified these relationships. The highest distress levels (50) were recorded when Negative Psychological Factors were at maximum intensity (1.0) and Positive Psychological Factors were absent (0.0). Conversely, distress was minimized (20) when Negative Psychological Factors were absent and Positive factors were at maximum intensity. The resulting surface plot illustrates that increasing positive psychological interventions significantly mitigates distress, even when negative factors are high, due to the non-linear interactions captured by the fuzzy model (Fig. 5).

## Discussion

**Fig. 6:**
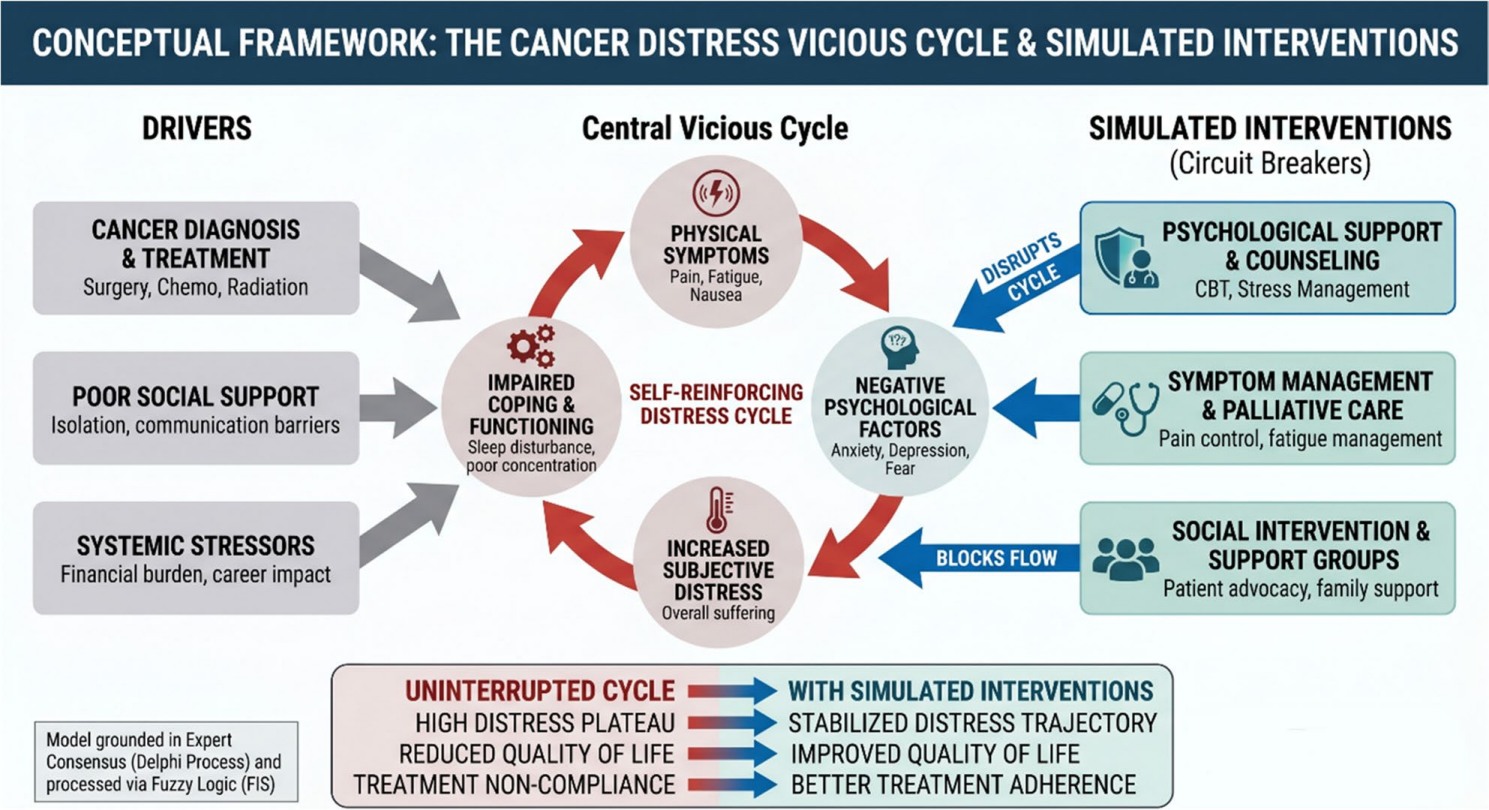
Conceptual Framework of the Cancer Distress Vicious Cycle and Simulated Interventions. Drivers (Left): External stressors—medical, social, and systemic—that trigger the initial distress response. Central Vicious Cycle (Center): A self-reinforcing loop where physical symptoms and negative psychological factors amplify subjective distress, further impairing patient coping. Simulated Interventions (Right): Clinical “circuit breakers” (e.g., CBT, symptom management, and support groups) designed to disrupt the distress cycle. Outcome Comparison (Bottom): Contrast between an escalating distress spiral and the stabilized plateau achieved through interventions. The framework is grounded in Delphi-derived expert consensus and processed via a Mamdani Fuzzy Inference System (FIS)

Our findings confirm the existence of a ‘vicious cycle’ in cancer distress—a self-reinforcing loop driven primarily by physical symptoms and negative psychological states. It is critical to note that this model is not purely theoretical; it provides the mathematical explanation for the interaction matrix that birthed the Distress Inventory for Cancer Version 2 (DIC-2). The DIC-2 has been rigorously validated in real-world cohorts exceeding 1000 patients and is a proven predictor of clinical outcomes, including treatment non-compliance and loss to follow-up [3, 4, 8–11, 13, 21, 22]. This finding aligns with established biopsychosocial models that view distress not as a static symptom, but as a dynamic process [6, 12]. By introducing inhibitory variables, such as Psychological intervention, Positive Psychological Factors and Conducive Environmental Factors, we demonstrated the potential to disrupt this cycle, consistent with literature emphasizing the role of social support and psychological interventions [23, 24]. The fuzzy model effectively captured the uncertainty in expert judgments, offering a robust alternative to crisp system dynamics models [17, 18] (Fig. 6).

### The advantage of fuzzy logic

The methodological novelty of this work lies in the deliberate synthesis of the Delphi process, network analysis, and fuzzy logic to resolve a classic clinical paradox: dynamically modeling a self-reinforcing loop using highly qualitative, subjective expert insights under sparse data conditions. As systematically contrasted in Table 6, traditional singular frameworks (such as linear regression or structural equation modeling) fail to capture these feedback loops or require large empirical datasets that render them unfeasible for expert-driven matrices. The fuzzy logic approach offered a robust alternative to traditional “crisp” system dynamics models by effectively capturing the uncertainty inherent in expert subjective judgments [6, 7]. While the crisp model showed a rapid, linear escalation of distress, the fuzzy model produced smoother, non-linear trajectories. This non-linearity is a more accurate representation of the human experience, where emotional responses do not always follow a straight line [1, 23]. The 25% reduction in distress observed in the fuzzy intervention case highlights the significant impact that even moderate psychological support can have on a patient’s overall state [2, 17].

**Table 6:** Comparative Analysis: Fuzzy Logic vs. Alternative Models

Modeling Technique	Analysis of Matrix Data	Resulting Differences & Limitations
Linear Regression / Correlation	Evaluates dyadic, one-way relationships.	**Misses the “Vicious Cycle”**: These models cannot capture feedback loops (e.g., Distress ◊ Symptoms ◊ Distress). They would provide a static “snapshot” rather than the dynamic, self-reinforcing system your data describes.
Structural Equation Modeling (SEM)	Attempts to fit the matrix to a predefined theoretical path.	**Requires “Big Data”**: SEM typically requires large empirical datasets to reach statistical power. Analyzing an expert matrix (*n* = 8) with SEM would lead to “under-identified” models with low reliability, whereas FL excels with sparse, expert-driven data.
Crisp System Dynamics (ODE)	Uses precise differential equations to model the weights.	**Extreme**,** Unrealistic Peaks**: Our results shows that the “crisp” model leads to rapid, near-linear growth (Distress levels jumping from 0.5 to 40). It lacks the “linguistic dampening” of FL, making the results mathematically sound but clinically implausible.
Machine Learning (ML)	Uses algorithms to find patterns in the matrix weights.	**Lack of Interpretability**: ML is a “black box” that requires massive training data to avoid over-fitting. In this context, there was no “ground truth” to train an ML model. FL is superior because it is auditable, clinicians can see the “If-Then” rules.

### Comparative analysis with existing tools

Compared to established tools like the NCCN Distress Thermometer [1] or the Distress Inventory for Cancer (DIC-2) [11, 22], our model provides a more nuanced view of inter-variable relationships, however, our findings are simulation-based proof-of-concepts and need to be verified in real world patient data.

Compared to alternative modeling techniques, the fuzzy logic approach is superior for the psycho-oncology domain. While ‘crisp’ system dynamics models often exhibit extreme, near-linear growth that lacks clinical realism, the fuzzy framework honors the ‘vague’ boundaries of emotional experience, resulting in stabilized trajectories that more accurately reflect patient satiation and response to intervention. By identifying ‘circuit breakers’, such as early psychological support, we provide a computational rationale for multidisciplinary interventions that can stabilize the distress plateau and improve quality of life.” In contrast, our fuzzy logic approach enables a nuanced representation of the distress cycle even with sparse data, making it particularly suited for the subjective nature of psychosocial oncology [17, 18, 25].

### Clinical implications

The model’s findings provide computational support for early psychological intervention. By specifically targeting negative psychological factors and symptoms early in the treatment trajectory, clinicians can integrate psychosocial support to enhance coping mechanisms. This approach aligns with NCCN guidelines and suggests that a multidisciplinary focus on environmental and psychological “inhibitors” can significantly improve a patient’s quality of life.

### Limitations and future directions

Despite its strengths, this study has limitations. The low response rate (eight experts) in the Delphi process may affect the generalizability of the interaction matrix. Furthermore, simplifying the fuzzy model to three primary variables might overlook secondary influences like the Time Factor. Future research should aim to:

- Incorporate more diverse expert cohorts to refine the interaction weights.
- Validate the model using empirical patient data to bridge the gap between expert consensus and clinical reality [15, 26].
- Integrate dynamic feedback loops to capture indirect effects over longer treatment durations [10, 22].

## Conclusion

This study provides a computational re-evaluation of the foundational expert consensus that established the interaction matrix for the Distress Inventory for Cancer (DIC) series. By integrating qualitative expert insights with the mathematical rigor of a Mamdani Fuzzy Inference System (FIS), we have elucidated the non-linear, self-reinforcing nature of patient distress.

Our simulations support the hypothesis that distress functions as a “vicious cycle” primarily propelled by physical symptoms and negative psychological states. A key methodological contribution of this work is the demonstration that a fuzzy logic framework captures the inherent linguistic ambiguity of clinical judgment more effectively than traditional “crisp” system dynamics models. The model demonstrated a simulated theoretical 25% reduction in distress when positive psychological and environmental interventions were applied as “circuit breakers” to the system.

While these findings are exploratory and limited by the historical nature of the Delphi sample, they provide the necessary mathematical rationale for the clinical success of the DIC-2, a tool that has been rigorously validated in cohorts and proven to predict treatment non-compliance. This work underscores the necessity of early, multidisciplinary interventions and reinforces the shift toward treating distress as the “sixth vital sign” in oncology. Ultimately, this model serves as a foundational blueprint for the future development of AI-assisted, personalized psychosocial decision-support tools.

## Data Availability

No datasets were generated or analysed during the current study.
